# High Breakdown Voltage and Low Buffer Trapping in Superlattice GaN-on-Silicon Heterostructures for High Voltage Applications

**DOI:** 10.3390/ma13194271

**Published:** 2020-09-25

**Authors:** Alaleh Tajalli, Matteo Meneghini, Sven Besendörfer, Riad Kabouche, Idriss Abid, Roland Püsche, Joff Derluyn, Stefan Degroote, Marianne Germain, Elke Meissner, Enrico Zanoni, Farid Medjdoub, Gaudenzio Meneghesso

**Affiliations:** 1 Department of Information Engineering, University of Padova, 35151 Padova, Italy; tajalli@dei.unipd.it (A.T.); zanoni@dei.unipd.it (E.Z.); 2 Fraunhofer Institute for Integrated Systems and Device Technology IISB, 91058 Erlangen, Germany; Sven.Besendoerfer@iisb.fraunhofer.de (S.B.); elke.meissner@iisb.fraunhofer.de (E.M.); 3 IEMN-CNRS, 59652 Villeneuve d’Ascq, France; riad.kabouche@ed.univ-lille1.fr (R.K.); idriss.abid.etu@univ-lille.fr (I.A.); farid.medjdoub@iemn.univ-lille1.fr (F.M.); 4 EpiGaN, 3500 Hasselt, Belgium; puesche@googlemail.com (R.P.); joff.derluyn@epigan.com (J.D.); stefan.degroote@epigan.com (S.D.); Marianne.Germain@epigan.com (M.G.)

**Keywords:** GaN, high-electron-mobility transistor (HEMT), trapping effect back-gating analysis

## Abstract

The aim of this work is to demonstrate high breakdown voltage and low buffer trapping in superlattice GaN-on-Silicon heterostructures for high voltage applications. To this aim, we compared two structures, one based on a step-graded (SG) buffer (reference structure), and another based on a superlattice (SL). In particular, we show that: (i) the use of an SL allows us to push the vertical breakdown voltage above 1500 V on a 5 µm stack, with a simultaneous decrease in vertical leakage current, as compared to the reference GaN-based epi-structure using a thicker buffer thickness. This is ascribed to the better strain relaxation, as confirmed by X-Ray Diffraction data, and to a lower clustering of dislocations, as confirmed by Defect Selective Etching and Cathodoluminescence mappings. (ii) SL-based samples have significantly lower buffer trapping, as confirmed by substrate ramp measurements. (iii) Backgating transient analysis indicated that traps are located below the two-dimensional electron gas, and are related to C_N_ defects. (iv) The signature of these traps is significantly reduced on devices with SL. This can be explained by the lower vertical leakage (filling of acceptors via electron injection) or by the slightly lower incorporation of C in the SL buffer, due to the slower growth process. SL-based buffers therefore represent a viable solution for the fabrication of high voltage GaN transistors on silicon substrate, and for the simultaneous reduction of trapping processes.

## 1. Introduction

Gallium nitride (GaN) high-electron-mobility transistors (HEMTs) on silicon (Si) substrate are excellent devices for power applications, thanks to the large bandgap, the high breakdown field strength, and the high electron saturation velocity of GaN [1,2]. Most commercial GaN devices are now targeting voltages up to 900 V, while other wide-bandgap semiconductors (such as SiC) offer viable solutions for voltage above 1 kV operation. Beyond-kV GaN devices would benefit from lower on-resistance than SiC technologies, while the growth on a silicon substrate would enable cost-effective device fabrication.

Recently, significant efforts have been deployed in order to find optimum GaN-on-silicon epitaxial structures enabling outstanding DC performance beyond 1 kV, while minimizing trapping effects [3,4,5,6]. There are two main issues that must be addressed: first, the vertical leakage must be minimized, to keep the off-state current of the devices below the safety limits. This requires a careful optimization of strain during growth, in order to minimize presence of defects like misfit dislocations, and an optimal compensation of the buffer via carbon doping. On the other hand, adding carbon to the buffer may result in undesired trapping effects, whose magnitude strongly depends on the vertical conductivity of the devices [7]. Viable strategies for reaching a few hundred volts on lateral GaN-on-Si HEMTs are: (i) partial substrate removal, that allows it to reach a vertical breakdown voltage above 1700 V. The main disadvantage of this technique is that it adds additional processing steps, not easily implementable at industrial level [3] (ii) the use of a resistive silicon substrate [8]; in this case, part of the vertical voltage falls on the depleted region in the silicon substrate, thus allowing us to significantly increase the vertical breakdown voltage. However, trapping effects must be carefully considered, as shown in recent papers [9].

Within this paper we demonstrate that high voltage operation with negligible trapping can be obtained through the use of a superlattice buffer, and that this permits the reduction of the thickness of the buffer, compared to a conventional step-graded approach. The study is motivated by the effectiveness of superlattice structures in lower voltage devices [10]. We compared an SL structure with a reference step-graded (SG) buffer (0.7 µm thicker than the SL one). Remarkably, the SL demonstrates a lower leakage current, especially at high voltages, which is ascribed to the better crystal quality of the buffer, confirmed by X-ray diffraction (XRD) measurements. In addition, the SL wafer shows a higher breakdown voltage and a much lower trapping. Substrate ramp measurements were used to obtain information regarding the dominant defect responsible for trapping up to T = 150 °C [11]. The results demonstrate that the trapping phenomena originate from the ionization of carbon acceptors on nitrogen site (C_N_) into the buffer [12].

## 2. Experimental Details

The study was carried out on two AlGaN/GaN heterostructures grown by metal organic chemical vapor deposition (MOCVD) on 1 mm highly conductive Boron-doped Si substrate with a diameter of 6 inches capped with a thick in situ SiN. The reference structure with a total thickness of 5.8 µm, consists of a 2.6 µm buffer (AlN nucleation layer and step-graded AlGaN layer), four AlGaN buffer layers with individual layer compositions and thicknesses tuned for optimal compressive strain generation, 2.9 µm carbon-doped GaN layer, a 0.3 µm unintentionally-doped GaN channel layer and 20 nm AlGaN barrier layer and a 50 nm thick SiN MOCVD passivation. A sheet resistivity of 300 Ω/sq has been measured with a mobility of 1800 cm^2^/Vs and an electron concentration of 1.15 × 10^13^ cm^−2^ for reference structure. The superlattice-based structure with a total thickness of 5.1 µm consists of an AlN nucleation layer, a 3.8 µm superlattice (140 periods of AlN/GaN layer pairs), a 1 µm carbon-doped GaN layer (the carbon concentration of the C-GaN grown on either buffer type was calibrated to be 10^19^/cm^3^), a 0.3 µm unintentionally-doped GaN channel layer, and an AlGaN barrier layer. The structure with SL shows a mobility of 1600 cm^2^/Vs and an electron concentration of 1.3 × 10^13^ cm^−2^. Both structures consist of Ti/Al/Ni/Au stack with 875 °C rapid thermal annealing, Ohmic contacts size is 95 × 95 µm^2^, have been formed on the top of the barrier by fully etching the thick in situ SiN cap layer using a Fluorine-based etching, yielding a typical contact resistance of 0.4 Ω·mm. Then, isolation around the contacts was performed by nitrogen ion implantation. The gate-source distance and the gate length are 1 µm and 2 μm, respectively.

**Figure 1:**
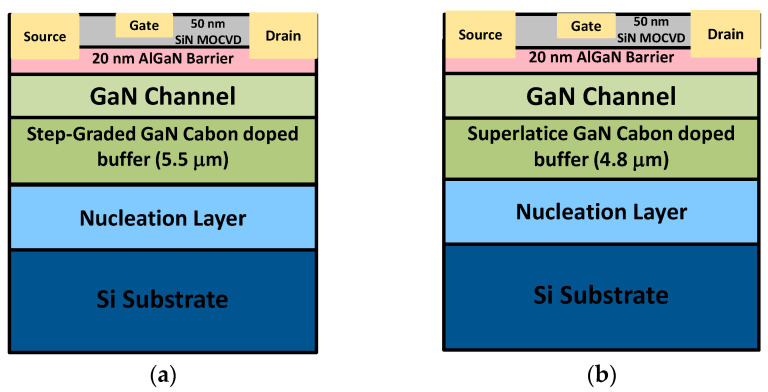
Schematic representation of the two tested samples. (**a**) The reference structure. (**b**) The superlattice-based structure.

Ni/Au gate metal was deposited within the in situ SiN by partly etching the cap layer with a 30 nm thickness as summarized in Figure 1. For investigating the leakage and breakdown, we carried out current-voltage characterization in the pico to milliamps range as a function of temperature. The trapping phenomena were investigated by substrate-ramp measurements, and by backgating drain-current transients [13]. Defect selective etching (DSE) was performed in a eutectic solution of KOH/NaOH at 450 °C for 4 min with sample backside protection and results were analyzed by a Jeol JSM-7500F scanning electron microscope (SEM, Tokyo, Japan). Panchromatic cathodoluminescence (CL) mappings were carried out at room temperature with a Gatan MonoCL3 system (Pleasanton, CA, USA) attached to the above-mentioned SEM.

## 3. Result and Discussions

### 3.1. Vertical Leakage and Breakdown

**Figure 2:**
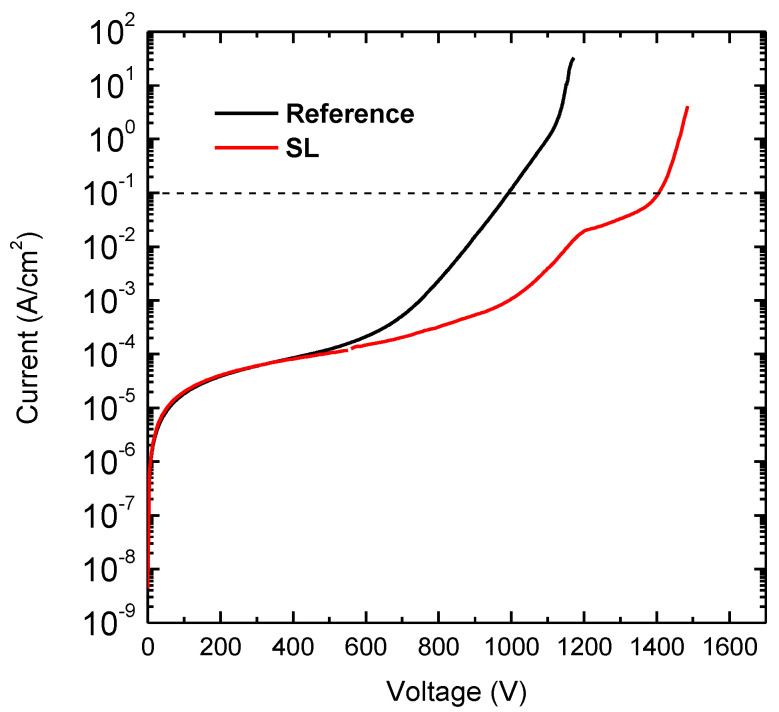
Vertical breakdown voltage at room temperature for the sample with and without superlattice (SL).

Vertical breakdown measurements were performed on both wafers by substrate grounded and Ohmic contact swept from 0 V up to breakdown voltage. At room temperature an average (soft) breakdown voltage of 1364 V at 100 mA/cm^2^ was reached for the structure with SL (standard deviation equal to 90 V), as compared to 980 V for the reference SG structure (standard deviation equal to 100 V). Figure 2 shows the typical breakdown characteristics of the two heterostructures. Electrical (hard) breakdown of the SL wafer was found to occur around 1500 V; this voltage corresponds to an average field of 3 MV/cm on the 5 µm vertical stack (under the simplified assumption that material properties and depletion are uniform across the various layers). This value is very close to the breakdown field of GaN (>3 MV/cm), indicating a good management of the field across the vertical stack.

**Figure 3:**
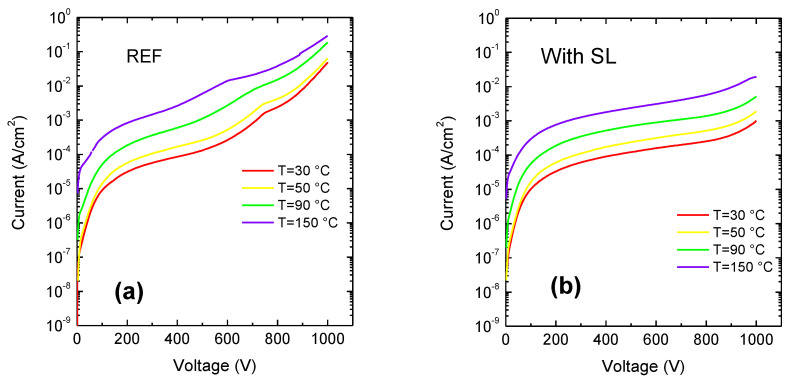
Vertical leakage current from room temperature up to T = 150 °C with (**a**) reference sample, (**b**) sample with SL.

**Figure 4:**
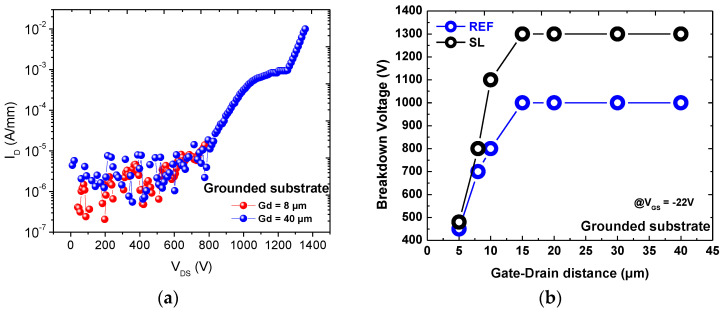
Three-terminal breakdown voltage with grounded substrate at room temperature (**a**) for device with SL and (**b**) for several gate-drain distances black curve (with SL) and blue curve (without SL).

Figure 3 reports typical leakage current characteristics measured at different temperatures for each wafer. As can be noticed, at high voltages the vertical leakage current of the SL wafer is one order of magnitude lower than that of the reference sample, up to 150 °C. This result might be ascribed to a better crystal quality due to a stronger strain mitigation by the use of a superlattice buffer [14,15,16]; also a higher energy barrier due to the SL can contribute. Three-terminal breakdown measurements have been carried out on HEMT. As can be seen in Figure 4a, devices with gate-drain (GD) distance of 40 µm deliver three-terminal breakdown voltage with grounded substrate around 1400 V, thus confirming that the developed material is viable for high voltage operation. The breakdown voltage (with grounded substrate) is much higher for the SL, compared to the reference buffer (Figure 4b) [17]. This proves the superiority of the SL buffer structure also in terms of device performance. Characterizations have been carried out on 2 × 50 μm transistors and gate length is 2 μm. To confirm the better epitaxial quality of the SL wafer, we carried out X-ray diffraction measurements. For asymmetrical plane GaN (102), the Full width at half maximum (FWHM) was 991 arcsec for the SL and 1216 arcsec for the reference wafer. The FWHM of GaN (102) is dependent on all types of threading dislocations (TDs), i.e., edge (TEDs), screw (TSDs) and mixed type and thus a measure for the overall structural quality of GaN [18]. The lower value found on the SL wafer confirms the better crystal quality of this structure.

**Figure 5:**
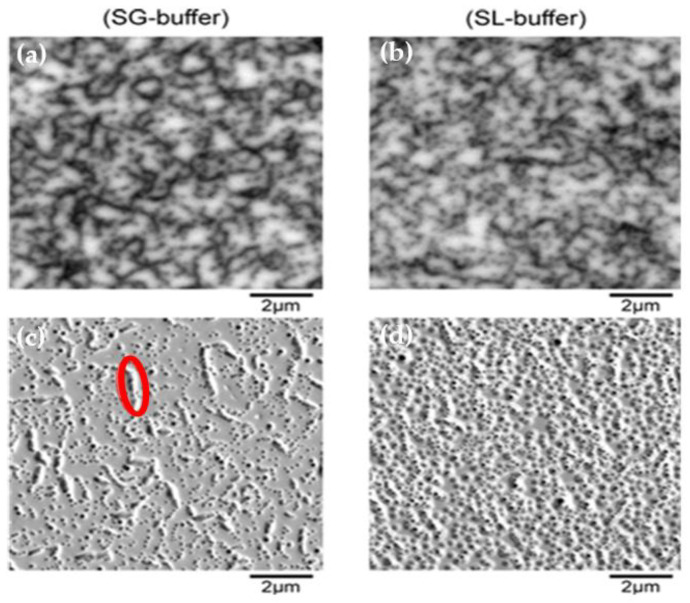
Representative CL mappings before defect selective etching (DSE) (first row) and SEM images collected after DSE (second row) on the two wafers (not the same place). On the step-graded (SG)-buffer sample extensive threading dislocation (TD)-clustering is observed (exemplarily marked by red ellipsis). (**a**) DSE on the reference wafer; (**b**) DSE on the SL wafer; (**c**) SEM image collected after DSE on the reference wafer; (**d**) SEM image collected after DSE on the SL wafer.

**Figure 6:**
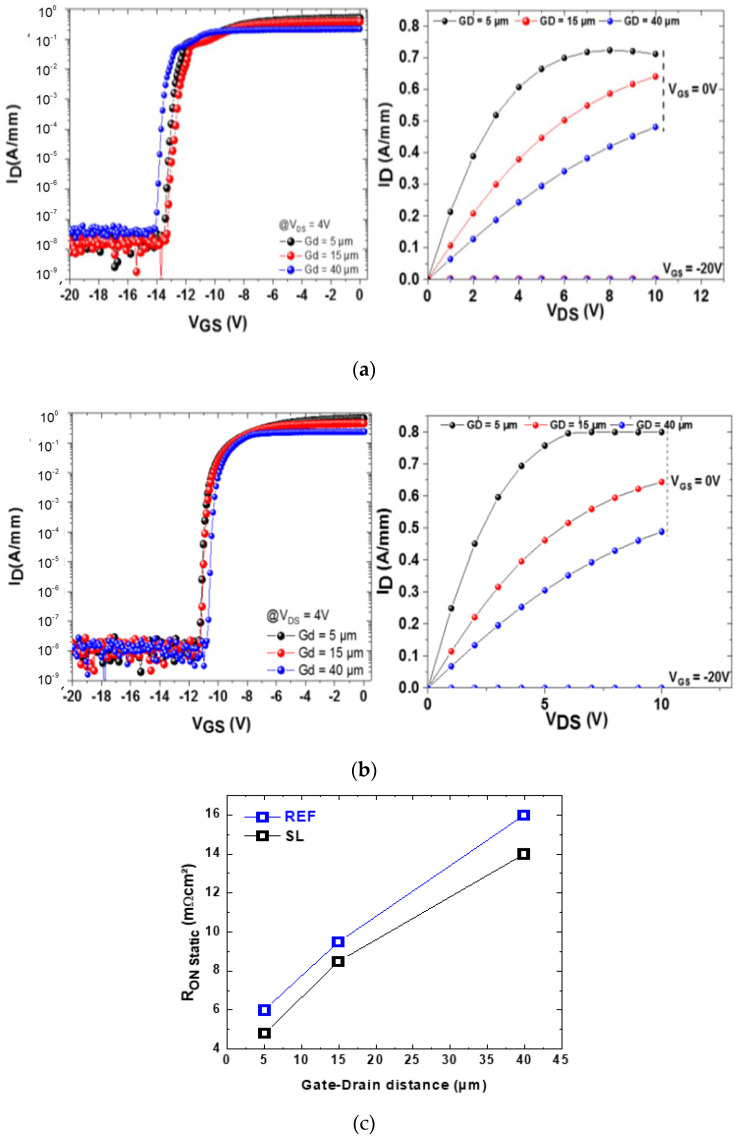
(**a**) Typical I-V characteristics for various gate-drain distances for REF structures. (**b**) Typical I-V characteristics for various gate-drain distances for SL structures. (**c**) On resistance for the SL and reference structure at different gate drain distance.

It is well known that TDs can favor vertical leakage conduction [19]. CL on the as-grown samples and DSE were performed in order to analyze density, areal distribution and partially the type of TDs at the AlGaN/GaN interface on a scale of 20 × 20 µm^2^ [20]. The result is shown in Figure 5. Additionally, panchromatic CL-mappings were performed after etching in order to verify that all TDs have been etched. Every pit represents a threading dislocation hitting the surface. The diameter of the pit is characteristic for the dislocation type. In terms of the total TD-density the samples are comparable: for the sample with the step-graded buffer, the total TD-density is 2.6 × 10^9^ cm^−2^ and for the sample with the superlattice buffer a slightly lower value of 2.3 × 10^9^ cm^−2^ was obtained by counting all pits in SEM images. The density of TSDs, a dislocation type that is often reported to be especially critical with regard to vertical leakage [21], is 2.5 × 10^7^ cm^−2^ for the step-graded sample and 3.7 × 10^7^ cm^−2^ for the superlattice sample. As mentioned, the total density of pits is slightly higher for the step-graded sample, but there is a significant difference in dislocation clustering. The step-graded sample shows extensive chain-like arrangements of TDs that are spaced only a few nm and correspond to TEDs [20]. The SL-buffer sample exhibits a more random distribution of TDs. According to the macroscopic measurements of vertical leakage we therefore assume that neither the total TD-density nor the TSDs play a dominant role in our case. The most important difference between the two wafers lies in the arrangement of TDs. This together with the slightly higher total TD-density, is reflected by the FWHM values of GaN (102) discussed above. TD-clustering, especially when present in the carbon-doped GaN layer underneath the channel layer, was assumed to be a potential issue for vertical breakdown by forming zones depleted in carbon [22]. As described above, the density of dislocations is comparable for the two wafers, while the difference lies in the degree of clustering of the dislocations. Figure 6a,b shows the I-V characterization of the HEMT devices which are similar for both Reference (REF) and SL devices. Characterizations have been carried out on 2 × 50 μm transistors and gate length was 2 μm. The measurements carried out on the HEMTs demonstrated that the on-resistance of the SL devices is comparable (and slightly lower) than that of reference samples, in Figure 6c. So, we conclude that the two-dimensional electron gas (2DEG) resistance is not affected by the type of buffer used.

### 3.2. Charge Storage in the Buffer

In order to evaluate the heterostructures in terms of charge trapping, we analyzed the charge storage in the buffer. Substrate ramp measurements were carried out on both SG and SL wafers in off-state condition, by ramping the bias from 0 V down to −800 V, −1000 V and −1200 V. The bias sweep is applied to the Si substrate, while a small bias of +1 V is also applied between two Ohmic contacts on the 2DEG on a transmission line measurement (TLM) structure, in order to highlight an eventual hysteresis reflecting the buffer charge trapping [7,11].

**Figure 7:**
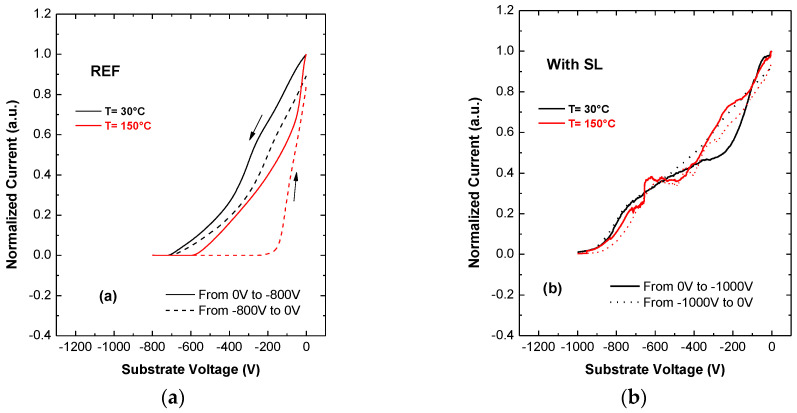
Substrate ramp measurements at room and high temperature. (**a**) Sample without SL, substrate voltage from 0 V to −800 V and (**b**) sample with SL, substrate voltage from 0 V to −1000 V.

Figure 7 reports the results of the measurements at room temperature (T = 30 °C) and high temperature (T = 150 °C) for the reference and SL wafers with 22.5 V/s sweep rate. The reference SG heterostructure shows low trapping effect down to −800 V at room temperature, while charge storage starts from −900 V (Figure 7a) negative substrate sweep induced a rightward shift of the curve, indicating a trapping of negative charge in the buffer. Charge storage is prominent also at 150 °C. The faster drop in current in the REF sample at 150 °C may be ascribed to a faster ionization of buffer traps. On the other hand, the optimized SL buffer shows a much lower trapping effect down to −1000 V (both at room temperature and T = 150 °C, see Figure 7b, and ensures state-of-the-art low trapping effects all the way down to 1200 V, not shown. The lower trapping of the SL buffer may also depend on the lower leakage current, that results in a lower availability of carriers.

To quantitatively evaluate the impact of charge trapping on drain current, and to understand why the reference and the superlattice buffer show different behaviors, we investigated the physical origin of buffer trapping by means of back-gating transients. The Backgating Current Transient (BCT) analysis can be employed to identify the trap origin in the buffer structure without the contribution of surface effects [23]: in fact, trapping is induced by a substrate pulse that only impacts on the vertical field. More specifically, the device under test is subjected to a trapping phase (V_D,F_; V_B,F_) in the off-state for a period of 10 s (here V_D,F_ and V_B,F_ are the bias points used on the drain and on the bulk to induce trapping). Then samples are biased in a low-field, low-power on-state in the de-trapping phase (V_D,M_; V_B,M_) for a period of 100 s to analyze the current (here V_D,M_ and V_B,M_ are the bias points used on the drain and on the bulk during the on-resistance measurement in the de-trapping phase). The measurements are carried out on TLM structures, allowing an accurate estimation of the impact of buffer trapping on the 2DEG resistance, the sampling time is 2 μs, 200 μs and 20 ms during the 100 s measurement.

**Figure 8:**
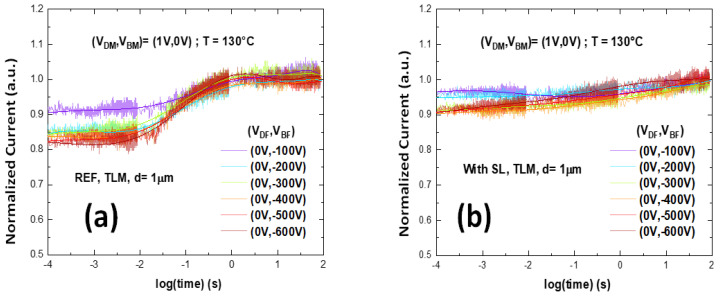
Back-gating transient at T = 130 °C on TLM with distance of 1 µm under six trapping conditions, (**a**) Sample A, reference device without SL, (**b**) Sample B, device with SL.

The BCT measurements were performed under six trapping conditions at T = 130 °C, in order to find the bias voltage which causes the trapping effect in the buffer structure. Right after the trapping bias is removed, current shows a gradual increase, related to the de-trapping of charge stored in the buffer. For the reference SG wafer, at 600 V initial current is almost 20% lower than in the de-trapped condition. For the SL wafer, initial current drop is less than 10%, indicating much less trapping. In addition, the de-trapping kinetics of the reference buffer show a dominant time constant, around 10–100 ms at 130 °C (Figure 8a), indicating the presence of a dominant trap. This is not found on the SL buffer (Figure 8b), where recovery kinetics are more gradual, as seen when there is no dominant trap, but bands of defects are present [24], or de-trapping is influenced by leakage [24]. 

In order to identify the defect responsible for charge trapping in the reference buffer (thus understanding why the SL buffer shows a better electrical behavior), we investigated the activation energy of the traps by temperature-dependent BCT measurements, under trap-filling bias conditions for 10 s and de-trapping conditions for 100 s (pulse conditions were (V_D,F_; V_B,F_) = (0 V; −300 V)).

**Figure 9:**
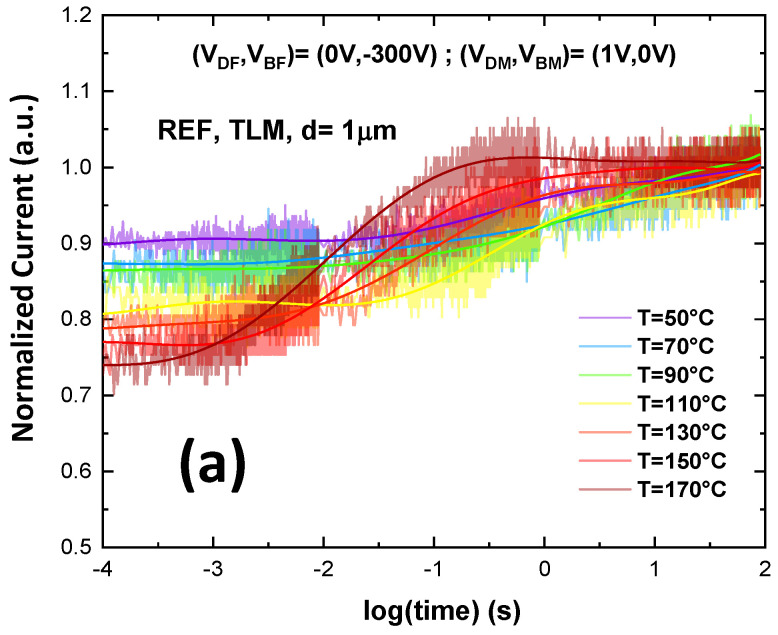

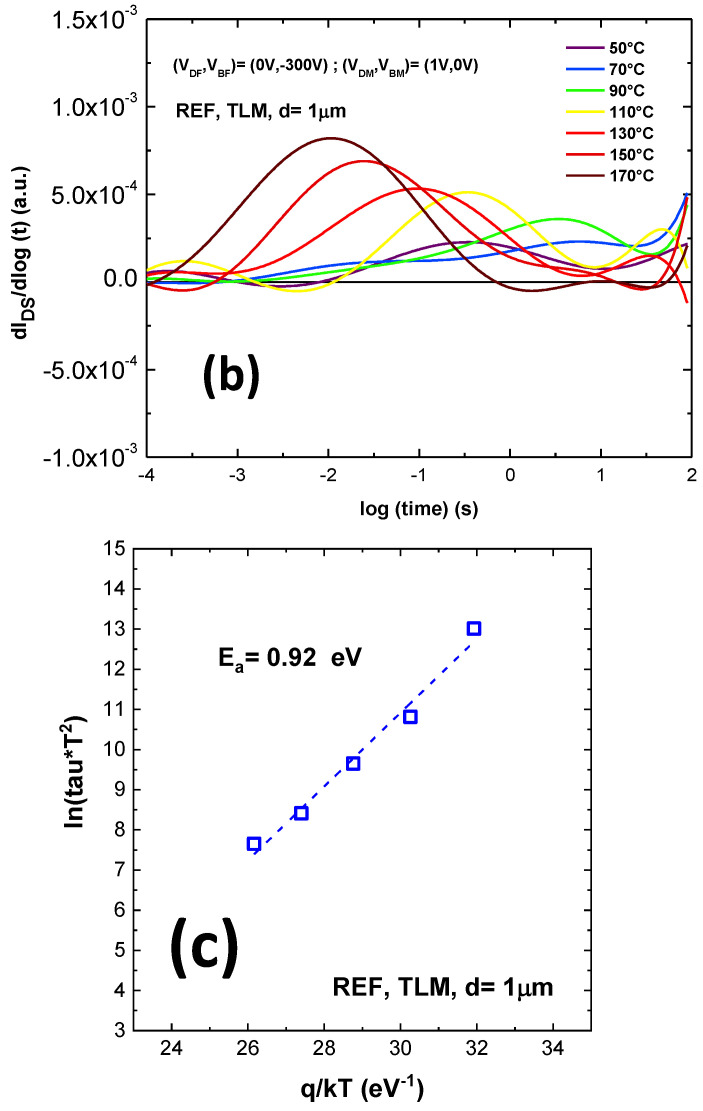
(**a**) Current transient behavior at multiple temperatures from 50 °C to 170 °C on a TLM with distance of 1 μm on the reference sample. (**b**) Time constant spectra corresponding to the current transient and (**c**) Arrhenius plot of the fitting time constants with activation energy of 0.92 eV.

**Figure 10:**
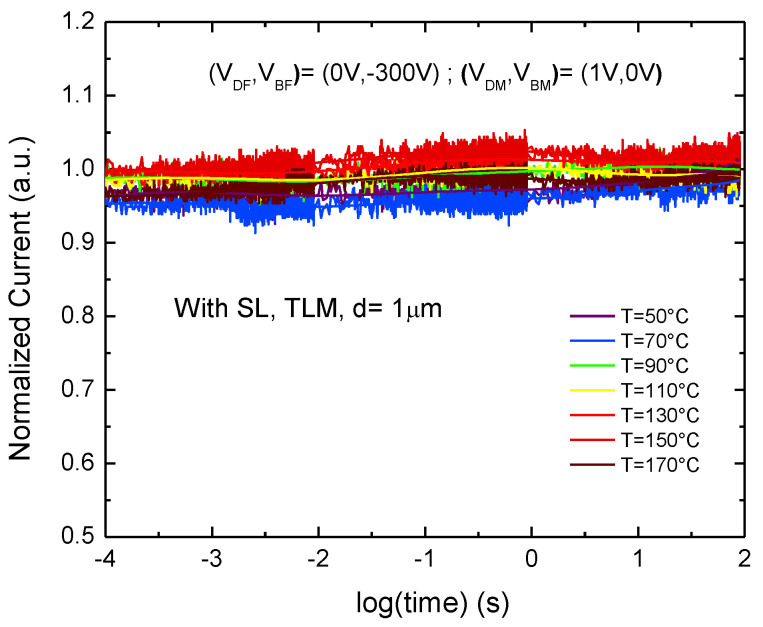
Current transient behavior at multiple temperatures from 50 to 170 °C on a TLM with distance of 1 μm on the sample with SL.

**Figure 11:**
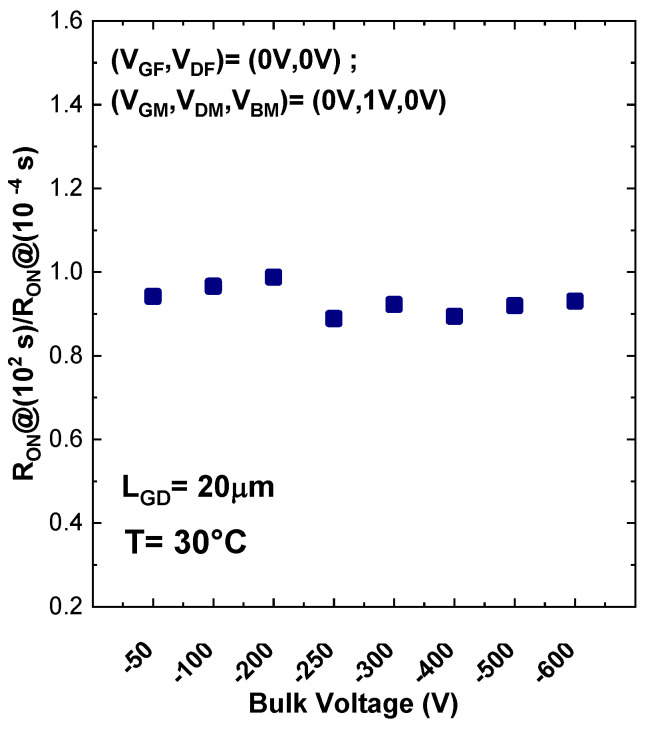
Normalized on-resistance at 100 s over on-resistance at 100 µs as a function of bulk voltage during the backgating measurement on the sample with SL. No significant variation was detected.

The current transient behavior at multiple temperatures on a TLM with distance of 1 µm is shown in Figure 9a. Moreover, Figure 9b reports the time constant spectra corresponding to the current transient in Figure 9a. To investigate the activation energy of the trap level in the buffer we did a linear fit of the time constants in an Arrhenius plot. The results of this measurement are summarized in Figure 9c, which indicates an activation energy of 0.92 eV, which is typically ascribed to the carbon acceptor, related to C_N_ located in the buffer [25]. On the other hand, the same measurement on sample B, including SL, shows that the trapping effect is completely eliminated thus the current is nearly flat even at high temperature as shown in Figure 10. Normalized on-resistance at 100 s over on-resistance at 100 μs on the sample with SL at different bulk voltage (from −50 V to −600 V) during the backgating measurement with LGD = 20 µm at room temperature shows no significant variation in Figure 11.

The fact that no relevant charging of C_N_ defects is observed for the SL buffer may be speculatively explained by the following considerations: (a) the nominal C-doping of GaN was identical for both the reference and the SL buffer. However, the growth rate of the SL wafer was slower than that of the reference structure, and this may have implied a lower carbon incorporation; (b) when submitted to high vertical bias, ionization of carbon acceptors is favored by vertical leakage. The lower leakage of the SL buffer may reduce the overall trapping effect at high bias, thus leading to a better dynamic behavior [15]; (c) the stronger chain-like clustering of TDs observed for the SG buffer sample is indicative for local clustering of TEDs at grain boundaries. TEDs can act as segregation centers for carbon and thus its concentration might be much higher very locally [26]. An inhomogeneous incorporation of carbon was generally shown to have a severe impact on dynamic device behavior [27].

## 4. Conclusions

In conclusion, we demonstrated that the use of a superlattice buffer can be a viable solution for the fabrication of high voltage GaN HEMTs with high breakdown voltage and low charge trapping effects. A 1500 V breakdown voltage was obtained with a 5 µm thick buffer, corresponding to an average field of 3 MV/cm, which is comparable to the theoretical breakdown field of GaN, indicating a good management of the electric field. An extensive comparison between a step-graded and a superlattice-based buffer indicated that the SL has superior performance thanks to (i) a higher crystalline quality in terms of less dislocation clustering, that results in a lower vertical leakage and higher breakdown; (ii) a possible lower incorporation of carbon, tentatively ascribed to a different growth rate, (iii) a lower leakage-induced charging of C_N_ and (iv) a lower presence of carbon-rich centers at clustered TEDs, both resulting in small charge storage.

The measurement results indicate that a proper buffer design along with the insertion of SL clears a way to GaN-on-silicon lateral power transistors at high voltage operation with very low trapping effects.

## References

[1] Amano H., Baines Y., Beam E., Borga M., Bouchet T., Chalker P.R., Charles M., Chen K.J., Chowdhury N., Chu R. (2018). The 2018 GaN power electronics roadmap. J. Phys. D Appl. Phys..

[2] Meneghini M., Meneghesso G., Enrico Z. (2017). Power GaN Devices.

[3] Dogmus E., Zegaoui M., Medjdoub F. (2018). GaN-on-silicon high-electron-mobility transistor technology with ultra-low leakage up to 3000 V using local substrate removal and AlN ultra-wide bandgap. Appl. Phys. Express.

[4] Tajalli A., Stockman A., Meneghini M., Mouhoubi S., Banerjee A., Gerardin S., Bagatin M., Paccagnella A., Zanoni E., Tack M. (2018). Dynamic-ron control via proton irradiation in AlGaN/GaN transistors. Proceedings of the 2018 IEEE 30th International Symposium on Power Semiconductor Devices and ICs (ISPSD).

[5] Herbecq N., Roch-Jeune I., Rolland N., Visalli D., Derluyn J., Degroote S., Germain M., Medjdoub F. (2014). 1900 V, 1.6 mΩ cm^2^ AlN/GaN-on-Si power devices realized by local substrate removal. Appl. Phys. Express.

[6] Umeda H., Suzuki A., Anda Y., Ishida M., Ueda T., Tanaka T., Ueda D. (2010). Blocking-voltage boosting technology for GaN transistors by widening depletion layer in Si substrates. Proceedings of the 2010 International Electron Devices Meeting.

[7] Uren M.J., Karboyan S., Chatterjee I., Pooth A., Moens P., Banerjee A., Kuball M. (2017). “Leaky Dielectric” Model for the Suppression of Dynamic Ron in Carbon-Doped AlGaN/GaN HEMTs. IEEE Trans. Electron Devices.

[8] Li X., Van Hove M., Zhao M., Bakeroot B., You S., Groeseneken G., Decoutere S. (2018). Investigation on Carrier Transport Through AlN Nucleation Layer From Differently doped Si Substrate. IEEE Trans. Electron Devices.

[9] Borga M., Meneghini M., Stoffels S., Li X., Posthuma N., Van Hove M., Decoutere S., Meneghesso G., Zanoni E. (2018). Impact of Substrate Resistivity on the Vertical Leakage, Breakdown, and Trapping in GaN-on-Si E-Mode HEMTs. IEEE Trans. Electron Devices.

[10] Stoffels S., Zhao M., Venegas R., Kandaswamy P., You S., Novak T., Saripalli Y., Van Hove M., Decoutere S. (2015). The physical mechanism of dispersion caused by AlGaN/GaN buffers on Si and optimization for low dispersion. Proceedings of the 2015 IEEE International Electron Devices Meeting (IEDM).

[11] Stockman A., Uren M., Tajalli A., Meneghini M., Bakeroot B., Moens P. (2017). Temperature dependent substrate trapping in AlGaN/GaN power devices and the impact on dynamic ron. Proceedings of the 2017 47th European Solid-State Device Research Conference (ESSDERC).

[12] Marso M., Wolter M., Javorka P., Kordoš P., Lüth H. (2003). Investigation of buffer traps in an AlGaN/GaN/Si high electron mobility transistor by backgating current deep level transient spectroscopy. Appl. Phys. Lett..

[13] Stockman A., Tajalli A., Meneghini M., Uren M.J., Mouhoubi S., Gerardin S., Bagatin M., Paccagnella A., Meneghesso G., Zanoni E. (2019). The Effect of Proton Irradiation in Suppressing Current Collapse in AlGaN/GaN High-Electron-Mobility Transistors. IEEE Trans. Electron Devices.

[14] Saito H., Takada Y., Kuraguchi M., Yumoto M., Tsuda K. (2013). Over 550 V breakdown voltage of InAlN/GaN HEMT on Si. Phys. Status Solidi Curr. Top. Solid State Phys..

[15] Meneghini M., Vanmeerbeek P., Silvestri R., Dalcanale S., Banerjee A., Bisi D., Zanoni E., Meneghesso G., Moens P. (2015). Temperature-Dependent Dynamic RON in GaN-Based MIS-HEMTs: Role of Surface Traps and Buffer Leakage. IEEE Trans. Electron Devices.

[16] Meneghini M., Tajalli A., Moens P., Banerjee A., Zanoni E., Meneghesso G. (2018). Trapping phenomena and degradation mechanisms in GaN-based power HEMTs. Mater. Sci. Semicond. Process..

[17] Albahrani S.A., Heuken L., Schwantuschke D., Gneiting T., Burghartz J.N., Khandelwal S. (2020). Consistent Surface-Potential-Based Modeling of Drain and Gate Currents in AlGaN/GaN HEMTs. IEEE Trans. Electron Devices.

[18] Papasouliotis G.D., Su J., Krishnan B., Arif R. (2015). (Invited) Epitaxial III-Nitride Film Growth in a Single Wafer Rotating Disk MOCVD Reactor. ECS Trans..

[19] Usami S., Ando Y., Tanaka A., Nagamatsu K., Deki M., Kushimoto M., Nitta S., Honda Y., Amano H., Sugawara Y. (2018). Correlation between dislocations and leakage current of p-n diodes on a free-standing GaN substrate. Appl. Phys. Lett..

[20] Besendörfer S., Meissner E., Lesnik A., Friedrich J., Dadgar A., Erlbacher T. (2019). Methodology for the investigation of threading dislocations as a source of vertical leakage in AlGaN/GaN-HEMT heterostructures for power devices. J. Appl. Phys..

[21] Hsu J.W.P., Manfra M.J., Lang D.V., Richter S., Chu S.N.G., Sergent A.M., Kleiman R.N., Pfeiffer L.N., Molnar R.J. (2001). Inhomogeneous spatial distribution of reverse bias leakage in GaN Schottky diodes. Appl. Phys. Lett..

[22] Knetzger M., Meissner E., Derluyn J., Germain M., Friedrich J. (2016). Correlation of carbon doping variations with the vertical breakdown of GaN-on-Si for power electronics. Microelectron. Reliab..

[23] Bisi D., Meneghini M., Marino F.A., Marcon D., Stoffels S., Van Hove M., Decoutere S., Meneghesso G., Zanoni E. (2014). Kinetics of Buffer-Related RON-Increase in GaN-on-Silicon MIS-HEMTs. IEEE Electron Device Lett..

[24] Uren M.J., Cäsar M., Gajda M.A., Kuball M. (2014). Buffer transport mechanisms in intentionally carbon doped GaN heterojunction field effect transistors. Appl. Phys. Lett..

[25] Lyons J.L., Janotti A., Van de Walle C.G. (2014). Effects of carbon on the electrical and optical properties of InN, GaN, and AlN. Phys. Rev. B.

[26] Wickenden A.E., Koleske D.D., Henry R.L., Twigg M.E., Fatemi M. (2004). Resistivity control in unintentionally doped GaN films grown by MOCVD. J. Cryst. Growth.

[27] Yacoub H., Zweipfennig T., Lükens G., Behmenburg H., Fahle D., Eickelkamp M., Heuken M., Kalisch H., Vescan A. (2018). Effect of carbon doping level on static and dynamic properties of AlGaN/GaN heterostructures grown on silicon. IEEE Trans. Electron Devices.

